# Microhardness and Fluoride Release of Glass Ionomer Cement Modified with a Novel Al^+3^ Complex to Enhance Its Antimicrobial Activity

**DOI:** 10.1155/2021/1925388

**Published:** 2021-10-23

**Authors:** Samy M. El-Safty, Nadia El-Wakiel, Gehan El-Oleimy, Mohamed Gaber, Yusif S. El-Sayed

**Affiliations:** ^1^Department of Dental Biomaterials, Faculty of Dentistry, Tanta University, Tanta 31527, Egypt; ^2^Department of Chemistry, Faculty of Science, Tanta University, Tanta 31527, Egypt

## Abstract

**Objectives:**

To synthesize and characterize a novel Al^+3^ complex with 2-(2-hydroxyphenyl)-1H-benzimidazole (HL) to be added to a restorative glass ionomer cement (GIC) to enhance its antimicrobial activities and to evaluate the Vickers microhardness (HV) and fluoride release (FR) of the modified GIC.

**Materials and Methods:**

Al^+3^ complex was synthesized by the addition of 1 mmol (0.210 g) of HL to 1 mmol (0.342 g) of aluminum sulfate in ethanol. The resulting solution was then refluxed under stirring for 24 h and then collected by filtration and dried in a vacuum desiccator over an anhydrous CaCl_2_. Characterization of Al^+3^ complex was carried out by Fourier transform infrared spectroscopy (FTIR), elemental microanalysis, thermal gravimetric analysis (TGA), molar conductance, ^1^H NMR spectra, and electron impact-mass spectrometry. The antimicrobial activity of Al^+3^ complex-modified GIC (Al-GIC) was studied by the “cut plug method” against Gram-negative bacteria (*Acinetobacter baumannii*) and Gram-positive bacteria (*Staphylococcus aureus*, *Enterococcus*, and *Streptococcus mutants*) and fungi (*Candida albicans*). HV was evaluated by a digital microhardness tester (Zwick/Roell, Indentec, ZHV*μ*-S, West Midlands, England). Fluoride levels in ppm were obtained using the ion-selective electrode connected to a digital meter. A one-way ANOVA and Bonferroni test were used to analyze the data with the significance level established at *p* ≤ 0.05.

**Results:**

Synthesis of Al^+3^ complex was confirmed by FTIR, elemental microanalysis TGA, molar conductance, ^1^H NMR spectra, and electron impact-mass spectrometry. Al-GICs exhibited an enhanced antibacterial activity against all studied microorganisms as confirmed by the growth of inhibition zones compared to control GIC (C-GIC). Though there was a slight reduction in HV and FR with increasing the added percent of Al^+3^ complex, no significant differences were found between the studied groups.

**Conclusions:**

Addition of Al^+3^ complex to GIC powder enhanced the antimicrobial activity of GIC materials. As there was a negligible insignificant reduction in HV and FR upon the addition of Al^+3^ complex, Al-GICs can be used with a guaranteed degree of clinical success.

## 1. Introduction

Because of their relatively desirable characteristics, such as fluoride release, chemical bonding, low coefficient of thermal expansion, adequate esthetics, and biocompatibility with the pulp tissues, dental clinicians have been using glass ionomer cements (GICs) in their daily dental practice [1, 2]. However, as these restorative materials suffer from some shortcomings, particularly mechanical properties, dental researchers have been modifying and developing new GICs for a reliable clinical use. Addition of resinous materials to produce resin-modified GICs (RM-GICs) represents one major modification that resulted in an increase in the mechanical properties and a lower solubility of GICs while maintaining their fluoride release and anticarious potential [3–5].

Considering them as important restorative filling materials, particularly in deciduous teeth, GICs were modified by the incorporation of antimicrobial agents. This addition enhanced the ability of GICs to resist development of secondary caries by inhibiting growth of invading bacteria [6, 7]. Polymers that contain quaternary ammonium salt (QAS) or quaternary phosphonium salt (QPS) were added to GIC powders and proved to have antimicrobial activities [8]. Furanone-modified GICs were also introduced and showed long-term antibacterial activities [9]. Propolis, also, was added to GICs and investigated. Studies revealed an antibacterial activity of propolis-GIC against the cariogenic bacteria, particularly *Streptococcus mutans* and *sobrinus* [10].

Chlorhexidine (CHX)—in the form of chlorhexidine gluconate, chlorhexidine acetate, or chlorhexidine hydrochloride—was incorporated into GICs and accepted by many authors because of its antibacterial activities against cariogenic bacteria [11]. Though affecting physical and mechanical properties and bonding capacity of GICs, CHX was reported to have a positive impact on fluoride release [12]. In the same direction, chitosan (CH) was used to modify GIC materials. Addition of 10% (by volume) of CH to GIC powder resulted in an increase in fluoride release and flexural strength of GIC, in addition to the antibacterial activity [13].

Benzimidazoles heterocyclic aromatic compounds are known to have an identical structure to that of the purine base of the DNA and the nucleus of vitamin B12. This similarity is very useful because it allows the biological systems to recognize the benzimidazole ring without any signs of allergic reactions [14]. These compounds are well known for their therapeutic properties, including antihypertensive, antihistaminic, antiulcer, antiviral, antifungal, and anticarcinogenic characteristics [15, 16].

**Figure 1:**
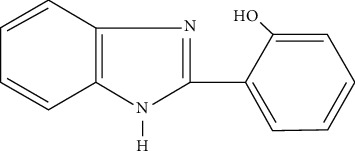
Chemical structure of 2-(2-hydroxyphenyl)-1H-benzimidazole.

A benzimidazole derivative, 2-(2-hydroxyphenyl)-1H-benzimidazole, has been reported to have a variety of medical applications. Investigations revealed that it has antibacterial, antifungal, anthelmintic, and antipoliovirus activities [17–19]. It is made up of the reaction of 1,2-dihydroxyphenol and salicylic acid [20]. The chemical structure of 2-(2-hydroxyphenyl)-1H-benzimidazole is shown in Figure 1.

The metal complexes of heterocyclic benzimidazoles and their derivatives have been receiving considerable attention in coordination chemistry. This is because of their well-documented biological activities [21]. As the earth's surface is profuse in aluminium (Al) compounds, conducting scientific researches utilizing such an element will be of great impact on our daily life [22]. When aluminum is joined to dyes, it produces less toxic and environmentally safer dyes [23]. Also, the aluminum complexes showed significant antifungal [24] and antibacterial [25] activities against a variety of microorganisms. In addition, Al^+3^ compounds have proven good mechanical properties where aluminum-based metal matrix composites have been widely used in different aspects of industrial products [26].

Despite the abundance of (Al) in the earth's crust and its usefulness in many applications, there have been few researches utilizing Al^+3^ complexes. That is why the current study will be focusing on synthesis of a novel Al^+3^ complex with 2-hydroxyphenyl benzimidazole (HL). After synthesis and characterization of such a new Al^+3^ complex, it was added to a restorative GIC to produce a modified GIC with improved antimicrobial activities. While preparing such a modified GIC, it is very essential to maintain adequate mechanical and physical properties. At the same time, it is very essential that fluoride ion-releasing capacity should not be affected by the addition of the synthesized Al^+3^ complex. Therefore, the main objectives of the current study were synthesis and characterization of a novel Al^+3^ complex, addition of this product to a well-known GIC to prepare a GIC with enhanced antimicrobial activity, and evaluation of the Vickers microhardness and fluoride ion release of the modified GIC. The null hypotheses of the study were as follows: (i) there will be no difference between the antimicrobial activity of control GIC and that of modified GICs and (ii) addition of Al^+3^ complex to the GICs will have no effect on the Vickers microhardness and fluoride ion release.

## 2. Materials and Methods

### 2.1. Materials

A commercially available glass ionomer cement (GIC) Fuji IX (glass powder and polyacrylic acid) (GC International, Tokyo, Japan) was used. 2-(2-Hydroxyphenyl)-lH-benzimidazole was purchased from Aldrich Chemical Company (Tokyo, Japan) and used as received. Aluminum sulfate was purchased from Aldrich Chemical Company (Tokyo, Japan) as well. Absolute ethanol was purchased from El-Nasr Pharmaceutical Chemicals Company (Cairo, Egypt).

### 2.2. Methods

#### 2.2.1. Synthesis of Al^+3^ Complex

The complex was prepared by the addition of 1 mmol (0.210 g) of HL to 1 mmol (0.342 g) of aluminum sulfate in ethanol. The resulting solution was then refluxed under stirring for 24 h. The chemical complex , which was separated while being hot, was collected by filtration and dried in a vacuum desiccator over an anhydrous CaCl_2_.

#### 2.2.2. Characterization of Al^+3^ Complex

*(1) Elemental Microanalysis*. The elemental microanalysis of (C, H, and N) contents of the Al^+3^ complex was carried out at the microanalytical center at Cairo University using PerkinElmer 2400 CHN Elemental Analyzer. A sample from the Al^+3^ complex with a mass of no more than 1 mg was burned in a vertical reactor (oxidation tube) in the dynamic mode at a temperature of about 1020°C in a helium (He) flow with the addition of O_2_ (10 ml) at the instant of sample introduction. The elemental analyzer software calculates the amounts of the resultant gases as a percentage of the initial sample weight. The results show %C, %H, and %N. In addition, these percents were calculated (mathematically) and the measured values were compared to those calculated [27].

*(2) Fourier Transform Infrared Spectroscopy (FTIR)*. Functional groups of HL and Al^+3^ complex were identified from the infrared spectra recorded using a JASCO FT/IR-4100 spectrophotometer. Samples from HL and Al^+3^ were prepared as KBr pellets by mixing 1 mg of each with 200 mg KBr powder. After thorough mixing with KBr, the mix was pulverized, put into a pellet-forming die, and pressed under a hydraulic press to form a tablet. The samples were scanned against a blank pellet background of KBr within the wavelength range of 4000–4000 cm^−1^ with a resolution of 4 cm^−1^.

*(3) Molar Conductance*. The molar conductance of complex solution was measured in dimethylformamide DMF (10^−3^ M)—a commonly used solvent in chemical reaction—at room temperature (25°C) using 523 conductivity bridge. This investigation provides a clue of the number of ions present in a particular solution responsible for the conduction of electric current and, thereby, quite significant structural information can be obtained. Molar conductance data are utilized to confirm electrolytic and nonelectrolytic nature of metal complexes.

*(4)* ^1^ *H NMR Spectra*. ^1^H NMR spectra were obtained using a Varian Mercury‐300BB NMR spectrophotometer operating at 300 MHz after dissolving the samples in DMSO‐d6 using tetramethylsilane as an internal standard.

*(5) Electron Impact-Mass Spectrometry (EI-Mass Spectrum)*. Standard electron impact mass spectrum (EI-MS) was determined using a Finnigan MAT 8222 Spectrometer at the microanalytical unit of Cairo University. It provides structural information and is considered as a “fingerprint” of the compound. The EI source is generally composed of a cathode (filament), an ion chamber, an electron receiver, and a set of electrostatic lenses. Under high vacuum conditions, a current is applied to the filament to emit electrons, and electrons are accelerated from the filament to the electron receiving end. In this process, the sample molecules collide with electrons in the ion chamber, causing the sample molecules to ionize or fragment into smaller parts. In order to stabilize the generated ion current, the energy of the electron beam is generally set at 70 eV, which results in a stable standard mass spectrum.

*(6) Thermogravimetric Analysis (TGA)*. Thermogravimetric analysis (TGA) of the solid sample was performed within a temperature range of 25–800°C using the Shimadzu TG-50 thermogravimetric analyzer with a heating rate of (10°C/min.) under a nitrogen atmosphere. This analysis determines the thermal stability/degradation of chemical compounds. Samples of Al^+3^ complex (2 mg) were gradually heated over a temperature range of 25–800°C and at a scanning rate of 20°C min^−1^ under a nitrogen atmosphere with a flow rate of 20 ml/min.

*(7) Molecular Modelling (MM+) Studies*. In an attempt to gain a better insight into the molecular structure of the metal complex, geometric optimization and conformational analyses were performed by the use of MM + force field as implemented in Chemistry Software, HyperChem Version 8, Molecular Modelling [28]. Semiempirical method PM3 was then used for optimizing the full geometry of the system using Polak–Ribière (conjugate gradient) algorithm. Unrestricted Hartree–Fock (UHF) theory is the most common molecular orbital method for open shell molecules where the number of electrons of each spin are not equal. Unrestricted Hartree–Fock theory uses different molecular orbitals for the *α* and *β* electrons. All the calculations refer to isolated molecules in vacuum.

#### 2.2.3. Formulation and Grouping of Al^+3^ Complex-Modified GICs (Al-GIC)

To prepare the experimental GIC groups (Al^+3^ complex-modified GICs), part of the GIC powder was replaced with a corresponding weight of Al^+3^ complex powder and thoroughly hand-mixed until a homogenous mixture of the two powders was obtained. Five groups were prepared: one control and four modified with the newly synthesized Al^+3^ complex. Al^+3^ complex powder was added to GIC powder at the percentages of 2.5%, 5%, 7.5%, and 10% to form the four modified (Al-GIC) groups, in addition to the control (C-GIC) group as follows:

  Group I (control): GIC without Al^+3^ complex
  Group II: 97.5 wt% of GIC mixed with 2.5 wt% Al^+3^ complex
  Group III: 95 wt% of GIC mixed with 5 wt% Al^+3^ complex
  Group IV: 92.5 wt% of GIC mixed with 7.5 wt% Al^+3^ complex
  Group V: 90 wt% of GIC mixed with 10 wt% Al^+3^ complex

#### 2.2.4. Screening of the Antimicrobial Activity

The antibacterial activity of Al^+3^ complex, C-GIC, and Al-GICs has been screened in vitro against Gram-negative bacteria (*Acinetobacter baumannii*) and Gram-positive bacteria (*Staphylococcus aureus*, *Enterococcus*, and *Streptococcus mutants*), and fungi (*Candida albicans*). The microorganisms were obtained from Microbiology Section, Botany Department, Faculty of Science. The bacterial strains were maintained and assayed on nutrient agar which contained 3 g peptone, 5 g NaCl, 5 g beef extract, and 20 g agar per litre. The antimicrobial activity of the tested samples was determined using “the cut plug method” [29]. Powder test samples (100 mg) of Al^+3^ complex, C-GIC, and Al-GICs were weighed. Wells were created in the agar plates and the powder was mixed with the liquid according to the manufacturer's instructions and then inserted into each well before setting to detect the most sensitive microorganisms. The plates were incubated at 37°C for 24 h; then the zones of bacterial growth inhibition were recorded in millimeters (mm) using a digital caliper.

#### 2.2.5. Testing of Vickers Microhardness (HV) of Al-GICs

Teflon molds were used to prepare disc-shaped specimens (*n* = 10) with a diameter of 8 mm and a thickness of 2 mm. A glass microscope slide, covered with transparent polystyrene matrix film, was positioned at the lower surface of the mold; then the GIC powder (Control and modified) was mixed with the polyacrylic liquid according to the instructions of the manufacturer. Within 60 s after the end of mixing, the mixed cement was packed to the molds with a slight overfilling. Another glass microscope slide, covered with transparent polystyrene matrix film, was positioned at the upper surface of the mold and a gentle hand-pressure was applied to extrude the excess material. When the specimen is completely set, excess material around the mold was removed by hand-grinding with 800 grit silicon carbide paper.

After removal from the mold, GIC specimens were stored in distilled water for 24 h before testing. Hardness testing was carried out using a Digital Microhardness Tester (Zwick/Roell, IDENTEC, ZHV*μ*-S, West Midlands, England) by applying a load of 2.9 N force for 15 s [30, 31]. Each specimen was fixed in a clamping apparatus and positioned in a manner that the indenter tip will be perpendicular to the specimen surface to be tested. Each specimen was subjected to five indentations equally spaced over a circle. Care was taken to make the indentation not closer than 1 mm to the adjacent indentations or the margin of the specimen. All results were generated and reported in HV units by using the microhardness tester. The Vickers hardness number (VHN) (kgf/mm^2^) for each tested specimen was measured according to the following equation [32]:

(1) $$\begin{array}{c} \text{VHN}=1.8544\times \frac{L}{d^{2}}, \end{array}$$

where *L* is the applied load (kgf) and *d* is mean diagonal length (mm).

Five indentations for each specimen were performed and averaged as the mean of that particular specimen. The mean of ten specimens was taken as the microhardness of the group.

#### 2.2.6. Fluoride-Release Measurement of Al-GIC

Ten disc-shaped specimens (*n* = 10) from each group were prepared in Teflon molds (8 mm in diameter and 2 mm in thickness). After complete setting, each specimen was immersed in 20 ml of deionized water in a closed plastic container at 37°C for 24 h. Measurement of fluoride ion was carried out by removing the specimen from its container and the storage solution was collected for analysis. After gentle drying of specimens, they were placed in a new container with fresh 20 ml of deionized water. The measurement of fluoride ion concentration was done using the ion-selective electrode method [31] that was applied for measuring at these periods: 24 h, 48 h, 1 week, and 1 month. Results were calculated as the amount of fluoride per unit surface area of the specimen (*μ*g/mm^2^). Fluoride levels in ppm were obtained using the ion-selective electrode connected to a digital meter. The total fluoride ion in *μ*g was calculated by multiplying the 1 ppm (1 *μ*g/ml) by the tested solution volume (20 ml). The total fluoride was then divided by the area of the tested specimen to obtain the fluoride release in *μ*g/mm^2^.

#### 2.2.7. Statistical Analysis

Data was collected and analyzed using an IBM compatible personal computer with SPSS statistical package version 20 (SPSS Inc.; released 2011; IBM Corp., Armonk, NY, USA). A one-way analysis of variance (ANOVA) followed by Bonferroni test was applied for analyzing the results of microhardness. As fluoride ion release measurement has two variables (time and percentage), two-way ANOVA was applied to examine the interaction between the two variables; then a one-way ANOVA was used for each variable separately. The significance level was established at *p* ≤ 0.05.

## 3. Results

### 3.1. Synthesis of Al^+3^ Complex

The synthesized Al^+3^ complex is white solid, stable even if it is exposed to moisture and air. It does not dissolve in alcohol but dissolves in dimethyl formamide (DMF) and dimethyl sulfoxide (DMSO).

### 3.2. Characterization Results of Al^+3^ Complex

#### 3.2.1. Elemental Microanalysis and Molar Conductance

The calculated values of C, H, N, and Al contents are in agreement with the found values which confirm the proposed molecular formulae (Table 1) for Al^+3^ complex. The molar conductance value in 10^−3^ M DMF (3 (Ω^−1^ cm^2^ mol^−1^) revealed the nonelectrolytic behavior of the metal chelate.

#### 3.2.2. Fourier Transform Infrared Spectroscopy (FTIR)

**Figure 2:**
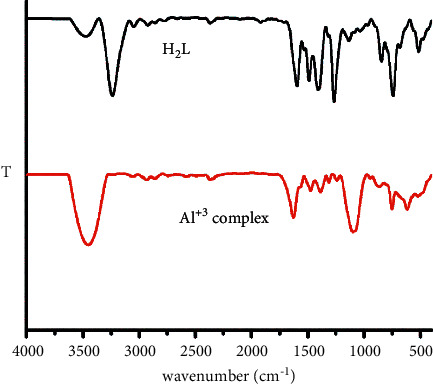
FTIR spectra of HL and its Al^+3^ complex.

As shown in Figure 2, the IR spectrum of HL showed 2 peaks at 3242 and 1608 cm^−1^ related to stretching vibration of the imidazole nitrogen (N-H and C=N). In the spectra of Al^+3^ complex, the position of the *ν*_N-H_ bands was minimally shifted to lower (6 cm^−1^) which confirms that the N-H group was not affected by the complex formation, but the band of *ν*_C_ _=_ _N_ was rather shifted to 1633 cm^−1^ due to the participation of C=N in chelation. The IR spectrum of HL showed bands at 1282 cm^−1^ related to the *δ*- OH group, which disappeared in the complex spectra, supporting the deprotonation of OH groups during complex formation.

The IR spectra of Al^+3^ complex showed a new band at 1235 and 1085 cm^−1^, which indicate the bidentate nature of sulphate ion [33]. Also, the IR spectrum of HL and Al^+3^ complex showed one peak at 3477 and 3454 cm^−1^*ν*_O-H_ of HL and water molecule of Al^+3^ complex, respectively. The Al^+3^ complex spectrum displayed new two bands at 618 and 470 cm^−1^ due to M-O and M-N bonds, respectively.

#### 3.2.3. EI-Mass Spectrum

**Figure 3:**
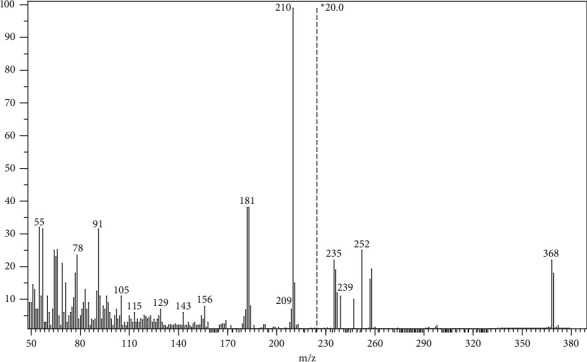
EI-Mass spectrum of the synthesized Al^+3^ complex.

Mass spectral analyses were used in order to assure the constituents of the synthesized complex. The mass spectrum of synthesized Al^+3^ complex showed the molecular ion peak at m/*z* 368.35 (Figure 3), which is equivalent to the molecular weight of the complex [ML]^+^. The appearance of this peak confirmed the formation of Al^+3^ complex.

#### 3.2.4. ^1^H NMR Spectra

**Figure 4:**
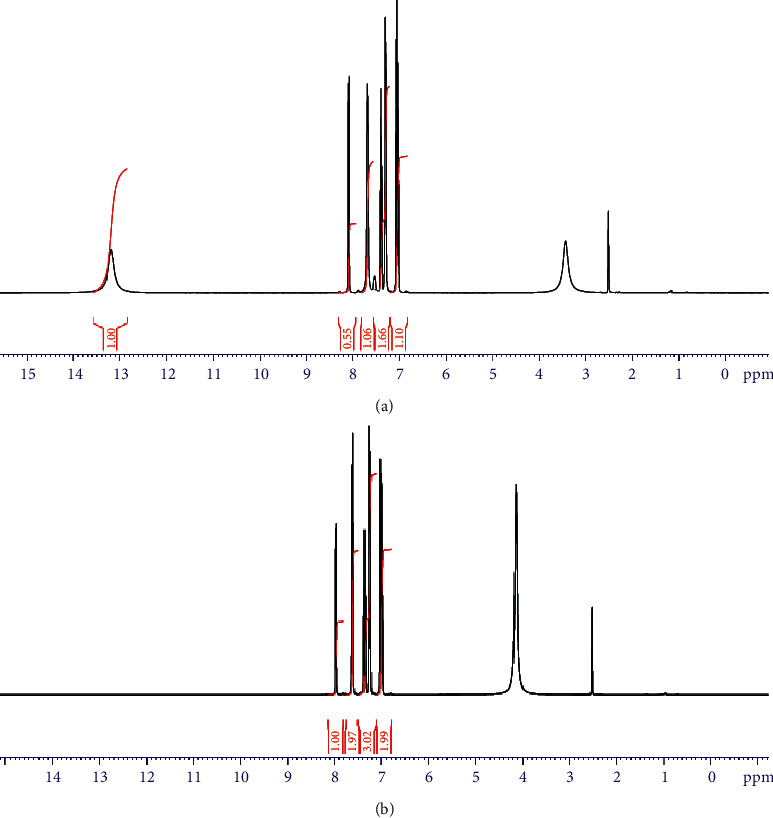
^1^H-NMR spectra of H_2_L. (a) DMSO. (b) D_2_O.

**Figure 5:**
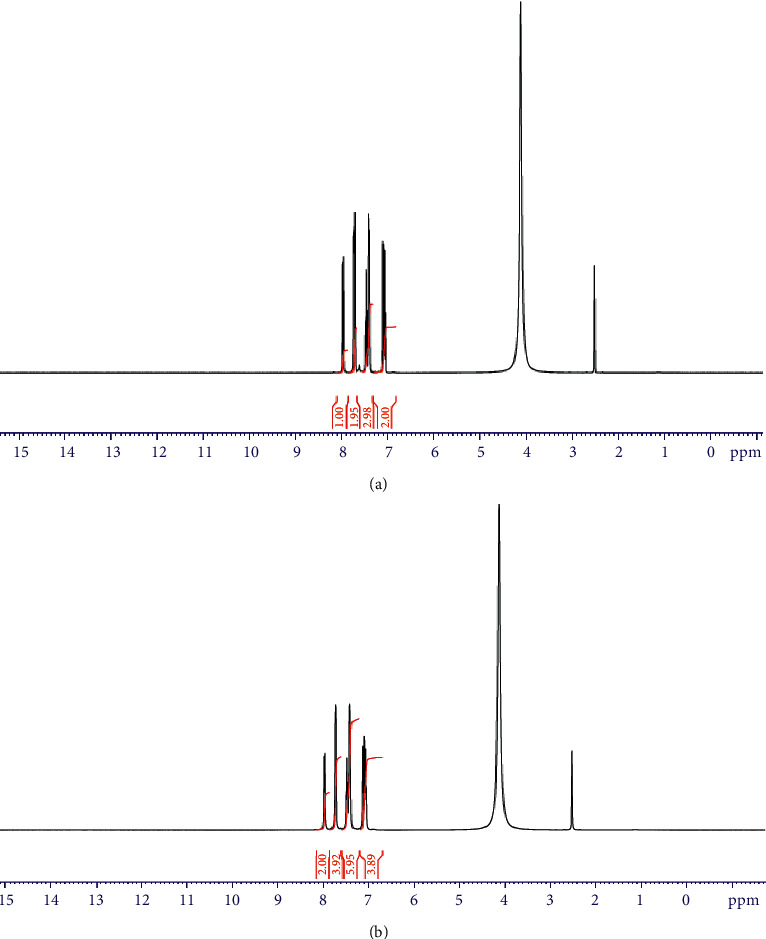
^1^H-NMR spectra of Al^3+^ complex. (a) DMSO. (b) D_2_O.

The ^1^H NMR spectra of the ligand and Al^+3^ complex were studied using DMSO and D_2_O as solvents. The spectrum of ligand (DMSO) displayed singlet peaks at 13.10 and 7.54 due to hydroxyl and N-H protons [34], respectively. These two signals disappeared after using D_2_O as a solvent (Figure 4). Also, ^1^H NMR spectrum of the ligand exhibited a multiple at 7.01–7.39 and 7.68–8.09 ppm which assigned to aromatic and benzimidazole protons. ^1^H NMR spectrum of Al^+3^ complex showed that the singlet peak of OH group was missing which conform the participation of OH group in complex formation (Figure 5).

#### 3.2.5. Thermogravimetric Analysis (TGA)

**Figure 6:**
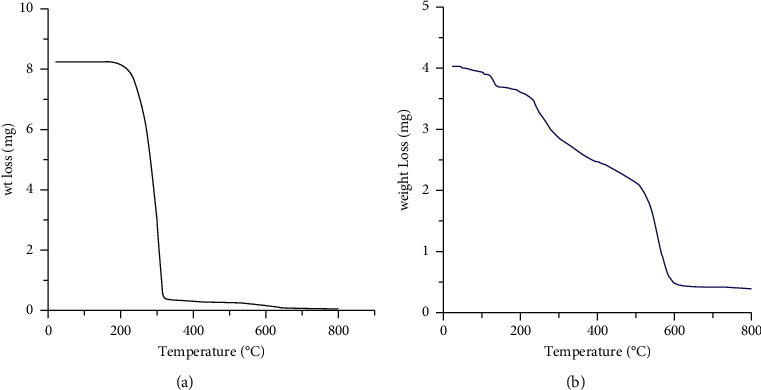
Thermal gravimetric analysis of HL (a) and Al^+3^ complex (b).

The TGA thermogram of HL displays thermal stability up to 200°C. The HL was completely depredated at 638°C. The thermogram of Al^+3^ complex showed thermal stability up to 90°C; after then the Al^+3^ complex gradually decomposed. The thermogram demonstrated three stepwise decomposition steps. The first decomposition step was at the range 90–179°C, and there was a weight loss of about 8.75% due to loss of coordinated water molecules. The second stage appeared at 179–362°C, and the weight loss of about 25.80% was because of removal of coordinated SO_4_^−2^ ion. The third decomposition step occurred within the range 362–613°C with a weight loss of 52.60%, representing the degradation of the organic ligand leaving Al_2_O_3_ as a final residue (Figure 6).

**Figure 7:**
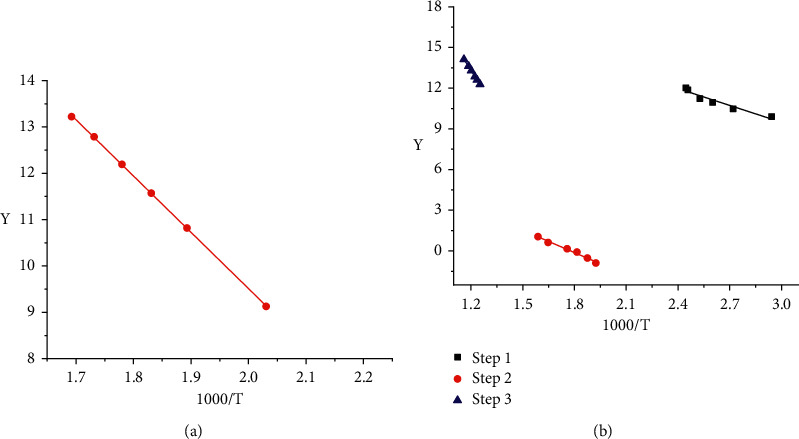
Coats–Redfern plots for the decomposition steps of HL (a) and Al^3+^ complex (b). Note: *Y*=[1 − (1 − *α*)^1−*n*^]/(1 − *n*)*T*^2^ for *n* ≠ 1 or *Y*=[−ln(1 − *α*)]/*T*^2^ for *n* = 1.

The kinetic parameters of activation energy (*E*), reaction order (*n*), and frequency factor (*A*) of complexes were evaluated using the integral method proposed by Coats–Redfern [35]. The results are listed in Table 2.  Figure 7 shows Coats–Redfern plots.

From the results listed in Table 2, the following features could be deduced: kinetics of thermal decomposition stages obeyed the 0.5 and zero to first-order kinetics. The negative values of the activation entropies *ΔS*^*∗*^ indicated a more ordered activated complex than the reactants and/or the reactions were slower than normal [36]. The high activation energy values (*E*^*∗*^) confirmed that the complexes under investigation are highly stable. The positive values of Δ*G*^*∗*^ suggested that this is a nonspontaneous process.

#### 3.2.6. Molecular Modeling

**Figure 8:**
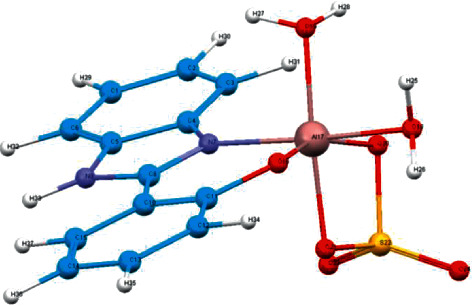
The fully optimized geometries of the synthesized Al^+^ complex.

The optimized structures, with atom labeling scheme, of Al^+3^ complex are represented in Figure 8. The study showed that the aluminum center is six-coordinated with NO donor set of the ligand and oxygen atoms sulfate group and water molecules, suggesting octahedral geometry around the metal center. The minimum and maximum cis angles around the metal center are 81.03° and 102.71°, while the minimum and maximum trans angles around the metal center are 156.91° and 175.52°. It is clear that the octahedral geometry is not much distorted. The obtained results are in agreement with the experimental results and, hence, strongly support them.

**Figure 9:**
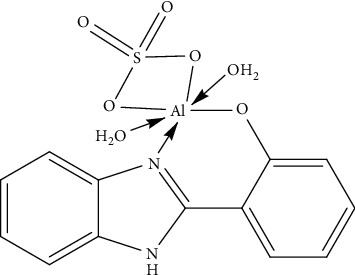
The proposed structure of the synthesized Al^+3^ complex.

Based on the FTIR, ^1^NMR, and EI-mass spectra results as well as the conductance values together with the data of elemental microanalysis and thermal gravimetric analyses, the Al^+3^ complex can be formulated as in Figure 9.

### 3.3. Antimicrobial Activity

**Figure 10:**
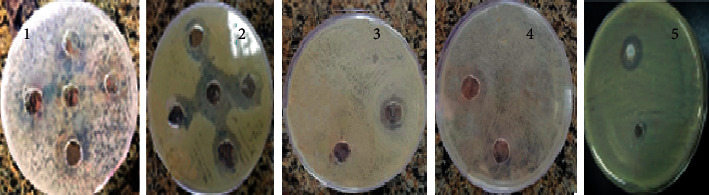
Antimicrobial activity (inhibition zones in mm) of Al^+3^ complex against the investigated microorganisms. 1: *Candida albicans*, 2: *Acinetobacter baumannii*, 3: *Staphylococcus aureus*, 4: *Enterococcus*, and 5: *Streptococcus mutans*.

**Figure 11:**
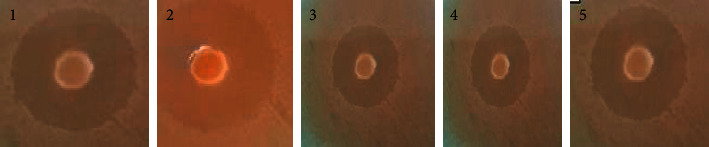
Antimicrobial activity (inhibition zones in mm) of Al-GIC (group V) against the investigated microorganisms.

The inhibition zones (in mm) of Al^+3^ complex (Figure 10) and Al-GICs (group V was taken as a representative, Figure 11) against the tested microorganisms are listed in Table 3. The data in Table 3 showed an antimicrobial activity of the ligand (HL) lesser than Al^3+^ complex and Al-GICs as well as C-GIC. Al^+3^ complex has a biological activity against all tested microorganisms, particularly *Acinetobacter baumannii* that recorded the best activity of Al^+3^ complex and modified Al-GICs.

### 3.4. Vickers Microhardness

Means and standard deviations of Vickers microhardness (HV) of C-GIC and Al-GICs are listed in Table 4. The highest HV was recorded for the control group (95.78) and the lowest value was shown by Al-GIC modified with 10% Al^+3^ complex (90.06). Statistical analysis showed no significant differences between studied groups. There was a systematic decrease (insignificant, *p*=0.732), compared to the control group, with increasing the percent of Al^+3^ complex.

### 3.5. Fluoride Ion Release

Fluoride ion release (FR) from all groups after immersion in deionized water for different times is shown in Table 5. All groups, control and modified, showed a reduction in FR over the immersion periods selected compared to the starting point (24 h). Generally speaking, over the four immersion periods (24 h, 48 h, 1 week, and 1 month), when the added Al^+3^ complex was increased in modified groups, there was a corresponding reduction in FR compared to C-GIC, with the least at 2.5% and the most at 10%. Statistical analysis showed no significant differences between studied groups of each chosen period (*p*=0.801).

## 4. Discussion

Continuous improvement of restorative dental materials has a great impact on the dental profession. This is because the addition of important characteristics to such materials enables them to possess beneficial properties and clinically serve in a better way. An example of this enhancement is applied in the current study, where a well-established GIC material was modified with a chemical compound to increase its antimicrobial activity.

As the metal complexes of heterocyclic benzimidazoles are well known for their desirable biological activities on the one hand and the well-established usefulness of Al ion on the other hand, a novel Al^+3^ complex with 2-hydroxyphenyl benzimidazole (HL) was synthesized and added to a well-marketed GIC material. The addition of such a complex was carried out at percentages (2.5–10%) that were thought to improve the antibacterial activity but not to deteriorate the other valuable properties of the restorative material. The antibacterial activity of the newly synthesized GIC was tested against some microorganisms of dental interest, such as *Candida albicans*, *Streptococcus mutans*, and *Staphylococcus aureus*, as well as against others to cover a wide range of microorganisms.

The first null hypothesis was rejected as all modified GIC groups reported better antimicrobial activity than the control group. Increasing the added percent of Al^+3^ complex recorded greater diameter of inhibition zones with all tested species. The greatest effect of the newly modified GIC was shown by *Acinetobacter baumannii* and the weakest was demonstrated by *Enterococcus*. Studies [37] reported that the antimicrobial mechanism of such chemical complexes can be one form or a combination of any of the following mechanisms: (i) adsorption onto the bacterial cell surface, (ii) diffusion through the cell wall, (iii) binding to the cytoplasmic membrane, (iv) disruption of the cytoplasmic membrane, (v) precipitation of cell contents and death of the cell, and (vi) release of potassium (K) ion and other cytoplasmic constituents.

However, for GIC materials in particular, there is a belief that the fluoride ions released from the GICs in the zone around them is the most probable reason for the inhibitory effect on acid production and there is a direct relationship between the fluoride released and the bacterial activity of the material [29]. The findings of the current study, however, demonstrated higher antimicrobial effect of the modified GICs compared to the control GIC group. Based on these findings and others [28], we can believe the critical role of the Al^+3^ complex in enhancing the antimicrobial activity of GIC materials.

As there were no statistically significant differences between the mean values of HV and FR among the studied groups, the second null hypothesis was accepted. The addition of Al^+3^ complex to GICs showed no detrimental effect on FR and HV. This can be considered a clinical benefit to the dental clinicians as the antimicrobial properties of the restorative material were enhanced without negatively influencing its vital properties.

Upon having a deeper insight into the results of both HV and FR of C-GIC and Al-GICs, it can be noticed that the addition of low percentages of Al^+3^ complex to GIC recorded negligible or very minute changes. However, increasing the percentage to 10% reported more visible, but insignificant, alterations in the means of the investigated properties. Generally speaking, the addition of a polymeric component may reduce the hardness of the GIC materials. However, in this study, the presence of Al ions in the added complex partly counteracted this reduction and the net result was a slight insignificant decrease of the hardness of the modified GICs compared to the control group. With regard to FR, the added complex did not influence much of the fluoride releasing capacity of GICs.

The synthesized Al^+3^ complex is white in color; therefore, its addition to the GIC powder will not change its desirable esthetic characteristics as a major requirement of the modern restorative dentistry. Such a modified GIC can be utilized in esthetic areas as a luting agent, provisional temporary filling, permanent filling of deciduous teeth, etc. The enhanced antimicrobial properties of the newly modified GIC hopefully can hinder the microbial ingress deeper under the restorations and prevent recurrent caries.

## 5. Conclusions

- Addition of the newly synthesized Al^+3^ complex to GIC powder improved the antimicrobial activity of GIC materials
- The insignificant decrease in HV and FR upon the addition of Al^+3^ complex to GICs may recommend the usage of such modified GICs with a guaranteed degree of clinical success

## Figures and Tables

**Table 1:** The elemental analysis, molar conductance data, and physical properties of the Al^+3^ complex.

Compound	Mol. wt	Color	Melting point (°C)	Elemental analysis % (found)				H_2_O%	SO_4_^−2^%
				C	H	N	Al		
Al^+3^ complex (AlLSO_4_ (H_2_O)_2_)	368.3	White	Over 250	42.39 (42.35)	3.56 (3.84)	7.61 (7.74)	7.33 (6.37)	9.77 (8.75)	26.08 (25.80)

**Table 2:** The kinetic and thermodynamic data of the thermal decomposition of HL and Al^3+^ complex.

Compound	Step	*n*	*r*	*E* ^*∗*^	Δ*H*	*A*	(−Δ*s)*	Δ*G*^*∗*^
HL	1	0.5	−0.99994	100.99492	96.145557	5.85*E* + 19	0.127925	170.7611

Al^3+^ complex	1	0	−0.96542	34.45	31.32	1.36*E* + 14	0.023747	40.25
	2	1	−0.99374	45.65	41.00	9.52*E* + 08	0.07825	2.76
	3	0.5	−0.99965	166.3	159.53	3.24*E* + 21	0.158518	288.68

**Table 3:** Antimicrobial activity (inhibition zones in mm) of HL, Al^+3^ complex, C-GIC, and Al-GIC (group V) against the investigated microorganisms.

Compound	Microorganism				
	Inhibition zones (mm)				
	Fungi	Gram −ve bacteria	Gram +ve bacteria		
	*Candida albicans*	*Acinetobacter baumannii*	*Staphylococcus aureus*	*Enterococcus*	*Streptococcus mutans*
HL	4	—	3	—	—
Al^+3^ complex	17	24	12	10	16
C-GIC	5	11	8	6	9
Al-GICs (group V)	14	19	10	7	13

**Table 4:** Means and standard deviations (in parentheses) of Vickers microhardness of C-GIC and Al-GICs.

Groups (% of Al^+3^ complex)	Microhardness (VHN)
Control group	95.78 (4.65)^a^
Group I (2.5%)	94.36 (3.64)^a^
Group II (5%)	93.17 (4.30)^a^
Group III (7.5%)	91.89 (3.28)^a^
Group IV (10%)	90.06 (2.29)^a^

Each value represents the mean of ten specimens. The same superscript letters indicate the absence of significant differences between groups (*p* ≤ 0.05).

**Table 5:** Fluoride ion release (*μ*g/mm^2^) from C-GIC and Al-GICs after immersion in deionized water for different times.

Groups (% of Al^+3^ complex)	Immersion time			
	24 hours	48 hours	1 week	1 month
Control group	0.152	0.122	0.119	0.115
Group I (2.5%)	0.149	0.118	0.117	0.115
Group II (5%)	0.146	0.116	0.115	0.114
Group III (7.5%)	0.141	0.113	0.112	0.112
Group IV (10%)	0.139	0.111	0.110	0.109

## Data Availability

The data used to support the findings of this study are included within the article. The authors do not have any restrictions to share these data with readers and researchers.
